# Interaction between Subjective Memory Decline and Depression Symptom Intensity in Older People. Results of the Second Wave of Cognition of Older People, Education, Recreational Activities, Nutrition, Comorbidities, and Functional Capacity Studies (COPERNICUS)

**DOI:** 10.3390/jcm10071334

**Published:** 2021-03-24

**Authors:** Sławomir Kujawski, Agnieszka Kujawska, Radosław Perkowski, Joanna Androsiuk-Perkowska, Weronika Hajec, Małgorzata Kwiatkowska, Natalia Skierkowska, Jakub Husejko, Daria Bieniek, Julia L. Newton, Paweł Zalewski, Kornelia Kędziora-Kornatowska

**Affiliations:** 1 Department of Hygiene, Epidemiology, Ergonomics and Postgraduate Education, Division of Ergonomics and Exercise Physiology, Collegium Medicum in Bydgoszcz, Nicolaus Copernicus University in Toruń, 85-094 Bydgoszcz, Poland; p.zalewski@cm.umk.pl; 2 Department of Geriatrics, Collegium Medicum in Bydgoszcz, Nicolaus Copernicus University in Toruń, 85-094 Bydgoszcz, Poland; agajos11@gmail.com (A.K.); perkowski.radoslaw@gmail.com (R.P.); joannaandrosiuk@gmail.com (J.A.-P.); weronika.topka.bydg@gmail.com (W.H.); malgorzata.gajos0904@gmail.com (M.K.); nataliaskierkowska1@gmail.com (N.S.); kubahusejko@gmail.com (J.H.); kasiakor@interia.pl (K.K.-K.); 3 Department of Physiology, Collegium Medicum in Bydgoszcz, Nicolaus Copernicus University in Toruń, 85-092 Bydgoszcz, Poland; 4 Department of Gastroenterology and Nutrition Disorders, Collegium Medicum in Bydgoszcz, Nicolaus Copernicus University in Toruń, 85-168 Bydgoszcz, Poland; daria.bieniek2@wp.pl; 5 Population Health Sciences Institute, The Medical School, Newcastle University, Newcastle-upon-Tyne NE2 4AX, UK; julia.newton@ncl.ac.uk

**Keywords:** cognitive function, older people, gerontology

## Abstract

Background: Prevalence of subjective memory impairment (SMC), with or without objective memory impairment, and the mediating role of depression symptom intensity was examined in older people. Methods: *n* = 205 subjects (60 years old and older) were examined and followed up at two years. Cognitive function was examined using the Montreal Cognitive Assessment (MoCA) Delayed Recall (DR) subtest. Geriatric Depression Scale (GDS) was used as a screening tool for depression. Statistical analysis was performed using linear mixed models. Results: A total of 144 subjects (70.24%) had SMC. MoCA Delayed Recall scores were not significantly changed in relation to time and SMC. Dynamics of SMC significantly influenced changes in GDS score (*p* = 0.008). Conclusions: SMC and objective memory impairment do not fully overlap each other. Subjects without SMC for longer than two years noted less intensity of depression symptoms in comparison to subgroup with SMC. However, occurrence of SMC in subjects who were previously free of SMC, was not related to increase in depression symptom intensity.

## 1. Introduction

Subjective memory complaints (SMC) form a core component of the criteria for Mild Cognitive Impairment (MCI) [1,2]. However, results of a meta-analysis on objective vs. subjective memory performance noted minimal correlation between objective performance and subjective assessments, with regards to memory function in general. This highlights the need for caution when only relying on the latter [3].

SMC could be related to depression in late life [4]. However, changes over time in this relationship needs further investigation. Results showed that SMC at baseline increased the risk of increased depressive symptom intensity after four [5] and ten years [6,7]. A recent analysis concludes that SMC in cognitively normal older people might contribute to the development of depressive symptoms in the future [4]. A subgroup with SMC were shown to be at a higher risk for developing depressive symptoms [4,5,6]. Findings from the English Longitudinal Study of Ageing show that in patients with objective cognitive impairment, depressive symptoms might show increased SMC, but this did not affect objective impairment [8]. In contrast, Lehrner et al. noted that depression is related to increase in SMC, regardless of objective cognitive function [9]. In summary, based on the results from previous studies, the relationship exacted between SMC, objective memory impairment, and depression severity, seemed to be unclear. A systematic review suggested that further studies examining relationship between those factors should be performed in a longitudinal manner [10].

Cognition of Older People, Education, Recreational activities, NutritIon, Comorbidities, fUnctional capacity Studies (COPERNICUS) is the first longitudinal study on aging in Poland [11]. A multidisciplinary approach is needed to examine the potential factors influencing cognitive function because of the well-described relationship between cognitive function and physical exercise [12], level of physical activity [13], and cognitive activity [14], in addition to traditional indicators of cognitive reserve, such as education level and occupational attainment [15]. In this study, we examined the prevalence of SMC, with or without objective memory impairment, and explored the mediating role of depression symptom intensity in older people.

## 2. Materials and Methods

### 2.1. Enrolment

**Figure 1:**
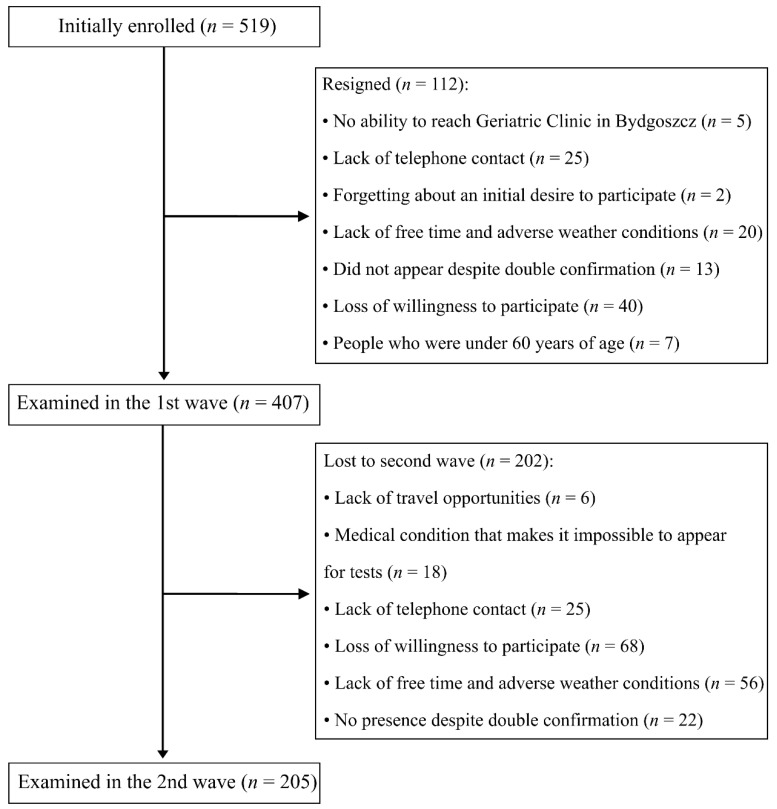
Flow chart of the study.

Figure 1 presents the process of study enrolment. In total, from *n* = 407 examined in the baseline cohort, *n* = 202 participants were lost to follow-up in the second wave of the study.

Participants were enrolled into studies based on advertisement, using regional TV and radio, during health-promoting lectures, in Day Care Centers for the Elderly, and at various meeting-groups for older people. Messages included information regarding an opportunity to a take part in a free-of-charge physical, physiotherapeutic, dietary, social, and cognitive assessment for people 60 years old and over. Age under 60 years old was the only excluding factor from participation in study. The lack of other factors excluding participation was underlined to collect the most representative sample of an older, Polish cohort, as much as possible. The study was approved by the Ethics Committee, Ludwik Rydygier Memorial Collegium Medicum in Bydgoszcz, Nicolaus Copernicus University, Torun (KB 340/2015, date of approval: 21 April 2015); and written informed consent was obtained from all participants.

### 2.2. Measurement

#### 2.2.1. Subjective Questionnaire

Subjective problems with memory were assessed twice—at the baseline and after 2-years follow-up. Data from both time-points was included in the statistical analysis. Participants were divided into subjective memory complainers (SMC) and non-subjective memory complainers (NSMC), depending on their answers to the question “Have You observed memory decline?” “Yes” was coded as “1”, “no” as “0”. At the 2-year follow-up, the time frame of this question was over the last 2 years. Then, the following answers on dynamics of memory decline could be chosen—“once it was better once worse”, “slow rate of memory decline”, “rapid”, and “it is hard to describe”. One of three answers on the presence of memory performance fluctuation during the day could be chosen—“yes”, “no”, or “it is hard to describe”. The time of day when the memory impairment could be described using one of the following options “worsen during the day”, “at the evening”, “at the morning”, “it is hard to describe”, and “none”.

#### 2.2.2. Cognitive Tests

Neuropsychological tests were conducted by two staff, who underwent common training in the procedures. Almost all (97.1%) neuropsychological tests were conducted by the same person. First, a questionnaire on subjective memory complaints was conducted. Cognitive functioning was assessed with the Mini-Mental State Examination (MMSE), Montreal Cognitive Assessment (MoCA), and Trail Making Test Part B (TMT B). MMSE is a well-known 30-points questionnaire used in neuropsychological assessment, it measures orientation to time and place, immediate recall, and short-term verbal memory, calculation, language, and construct ability. A higher score indicates better cognitive performance [16]. MMSE accuracy and efficiency seems to be worse than other screening tests aiming to measure cognitive function in older people [17]. However, it was widely used in previous research on older patients in Poland [18,19,20].

The MoCA assesses several cognitive domains [21,22]. It measures all main cognitive domains; namely visuospatial skills, short-term memory recall, executive functioning (examined by a mini-form of Trail Making Test part B, phonemic fluency task, and a two-item verbal abstraction task). Attention, concentration, and working memory, as well as naming and other language skills were evaluated. In MoCA, test results of two subtests (Verbal Fluency subtest and Delayed Recall of five nouns) were taken into account separately during analysis. The Verbal Fluency subtest result is the number of words in Polish starting with the letter “S”, which are not own nouns (conjugation prohibited). In the case of Delayed Recall, two score was taken into analysis—first was the number of words recalled without the help of person carrying out the test (MoCA Delayed Recall subtest). The second score was the overall number of words recalled without help, and number of words recalled after the category of the word recalled and the number of words were correctly chosen from a list of three words (MoCA Delayed Recall subtest overall score). Results from the Delayed Recall subtest served as an indicator of verbal short-memory performance. Result of this subtest was used to address the primary outcome of the study, namely the relationship between subjective and objective memory impairment with depression severity. The Polish version of MoCA was shown to be accurate in assessing the cognitive function level in previous research [23].

Trail Making Test part B is a fast-to-assess neuropsychological tool, which measures various skill from the executive functioning domain—visuospatial skills, task switching, and working memory, to mention a few [24].

#### 2.2.3. Emotional State Assessment

The 15-item Geriatric Depression Scale (GDS) is a self-report measure of depression dedicated to older patients’ assessment [25]. This shorter version was proven to be useful among very old people, with and without cognitive impairment [26]. Questions concerning assessment of retrospection, the quality of life, current state, activities, mental state, life attitude, and other questions were asked. Answers were presented in dichotomized (yes/no) format. The higher the score, the higher the severity of depression. GDS sensitivity and specificity was calculated as 84% and 95%, respectively [25,27].

#### 2.2.4. Functional Performance Assessment

A six-minute walk test (6MWT) was performed [28]. Eight feet up-and-go result is an indicator of functional performance, gait speed, and balance, in a dynamic manner. Subject was asked to get up from the chair and walk a distance of 8 feet, to and around a marker placed on floor, get back and sit on the chair again, as fast as possible [29].

#### 2.2.5. Activity Level Assessment

Answers on questions about the frequency of current physical, cognitive and social activities, and diet, were coded in the following way—“never” was coded as 0, “once a year” as 1, “several times a year” as 2, “1–2 times a month” as 3, “once a week” as 4, “few times a week” as 5, and “daily” as 6. The result of the questionnaire was the overall score from all questions. The following questions on cognitive and social activities were asked—reading press, reading books, watching TV, listening to the radio, going to the café, restaurant, going to the cinema, going to the theater or concert, going to church, going to visit friends or family, taking part in social group meetings, computer use, card game, chess/checkers, and solving crosswords. In addition, questions on the following physical activities—short walks around the house place, long walks, gymnastics, cycling, running/jogging, swimming, skiing, team games, sailing, horse riding, Nordic walking, tennis/table tennis, dance, and work on the plot or in the garden/mushroom collection. The following questions on frequency of travel activities in the last 3 years were asked: 1-day trips without accommodation, multi-day sightseeing trips, pilgrimages, wandering (with accommodation in hostels, campsites), stays at campsites, with accommodation, holiday/holiday stays, trips stationary, leisure trips to friends, stays on plot (outside your home), spa stays (prophylactically without referrals to the sanatorium), and stays in sanatoria on the basis of a referral. Frequency of consumption of beer, wine, vodka, and other strong alcohols was assessed. Overall frequency was the score of ethanol consumption. Financial status was calculated indirectly, based on particular items in an activity questionnaire (such as frequency of going to restaurants, traveling abroad etc.).

### 2.3. Statistical Analysis

All statistical analyses were performed using the statistical package R [30]. Mean and standard deviation (SD) values are presented. To assess the relationship of SMC with changes in objective short-term verbal memory decline (before vs. after two years) and GDS score, a linear mixed model with a restricted maximum likelihood approach, and *t*-tests (using the Satterthwaite method) was used. Analysis was carried out in R statistical packages (Lme4 and LmerTest packages was used [31]). The subject and time factors were determined as random effects. Plots were created to show the interaction between SMC and time (before vs. after 2 years). The vertical lines denote the 95% confidence interval. Post-hoc tests results were adjusted using the Holm method to counteract the problem of multiple comparisons using lsmeans and multcomp packages [32].

## 3. Results

### 3.1. Baseline Data from Those Participating in the Follow-Up Compared to Those Not Participating

Table 1 shows the comparison between baseline data from those participants lost to follow up in the second wave compared to those who were re-examined. The age of participants lost to follow-up were not significantly different as compared to those re-examined. Scores of MMSE, MoCA, and TMT B were significantly worse in subjects lost to follow-up. Moreover, the group of re-examined subjects who spent significantly more years in education, were characterized by higher cognitive, social, and overall activity level, as well as better financial status.

At baseline, subjective memory impairment was noticed by 73.8% of the subgroup lost to follow-up and by 79% of the re-examined participants. One hundred twenty-five subjects reported SMC at baseline and after 2 years. Thirty-seven subjects reported SMC at the baseline, however, were free of SMC after 2 years (Table 2).

### 3.2. Comparison of Measures in the Cohort with Baseline and Follow-Up Data

In the cohort of 205 re-examined subjects, 144 (70.24%) had SMC. Twenty subjects (13.99%) with SMC noted memory impairment fluctuation during the day. At the baseline, ninety-five participants had a history of arterial hypertension, of which 58 received antihypertensive treatment.

**Figure 2:**
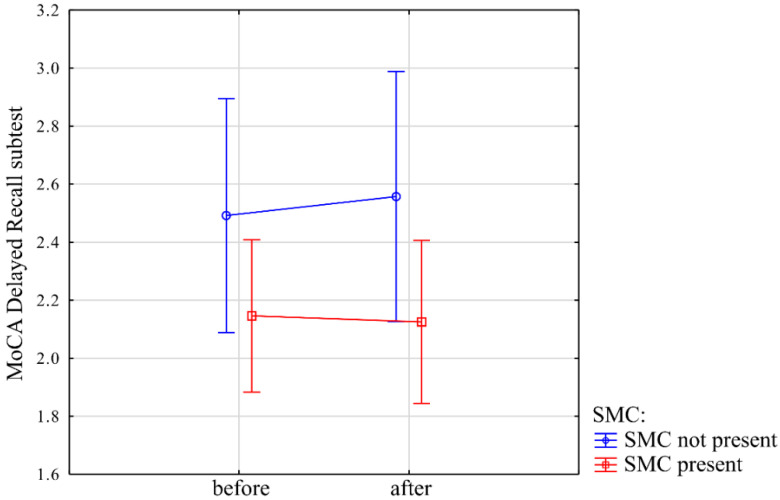
Interaction between time and subjective memory complaint (SMC) presence and results of MoCA Delayed Recall subtest. Y-axis (MoCA Delayed Recall subtest) represents the number of words recalled correctly in short-term verbal memory test in MoCA without the help of staff conducting the examination. X-axis (“before” and “after”) denotes the initial time-point and after two years, respectively. Blue line indicates subgroup without subjective memory complaints, while red line indicates scores of participants with subjective memory complaint.

Interaction between time and SMC factors in the results of MoCA Delayed Recall subtest score (which is the number of word recalled without the examiners help) was not statistically significant (−0.09 (−0.6; 0.4), *t* = −0.34, *p* = 0.73) (Figure 2).

**Figure 3:**
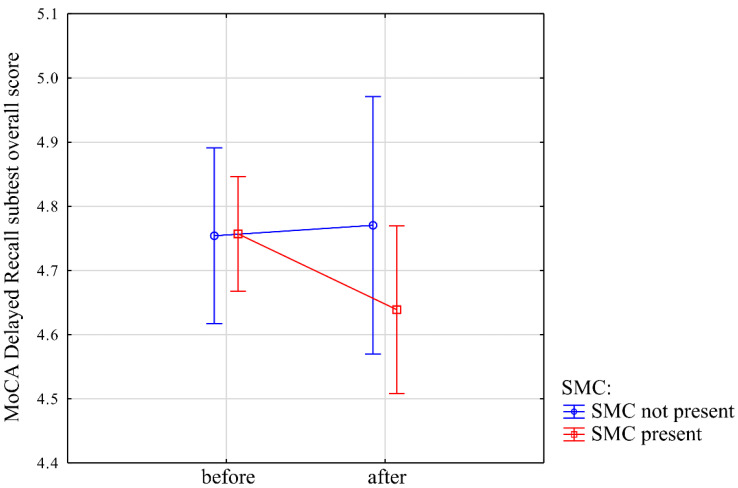
Interaction between time and subjective memory complaint (SMC) presence and results of the overall MoCA Delayed Recall subtest. Y-axis represents the number of words overall recalled correctly in the short-term verbal memory test in MoCA, after category, and the list cues. First, a category of word was recalled, then the participant was asked to choose the correct word from a list of three words from the same category. Sum of the overall number of words recalled by the participant themselves, with the category and list cue. X-axis (“before” and “after”) denotes the initial time-point and after two years, respectively. Blue line indicates a subgroup without subjective memory complaints, while the red line indicates scores of participants with subjective memory complaint.

Interaction between time and SMC and the overall results of the MoCA Delayed Recall subtest was not statistically significant (−0.14 (−0.39; 0.11), *t* = −1.01, *p* = 0.31) (Figure 3).

The interaction between time and SMC and the other tests (MMSE, MoCA, TMT B) were also non-significant. Participants with SMC in the second wave (follow up) were significantly older, had worse TMT B, showed higher depression severity, a slower gait, and a worse aerobic capacity compared to subgroup without SMC in the baseline (Table 3).

Subjects with SMC and objective verbal memory decline in the second wave had significantly lower years of education, better MoCA, Delayed Recall subtest scores, and worse TMT B performance, as compared to subgroup without subjective and objective memory impairment in the baseline (Table 4).

**Figure 4:**
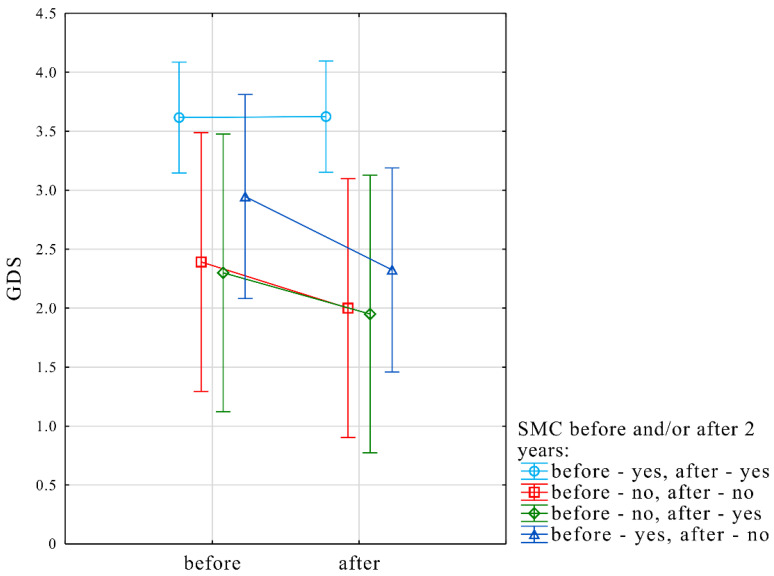
Results of linear mixed model predicting GDS score change over two years in relation to the presence of subjective and objective verbal memory decline. Y-axis represents the score on the Geriatric Depression Scale (GDS). X-axis (“before” and “after”) denotes the initial time-point and after two years, respectively. Light blue line indicates the subgroup of subjective memory complaint (SMC) at both time-points, red line indicates subgroup without SMC at both time-points. Green line indicates participants who were free of SMC at the initial time-point, but it changed after two years (after). Dark blue line denotes subgroup with SMC at the initial time-point and who was free of SMC after two years (after).

GDS was included as a dependent variable with SMC, before and after 2 years as a fixed effect, while subject and time factors were set as random effects. Dynamics of SMC was significantly related to changes in GDS scores (F = 4.9, *p* = 0.008). Participants who did not report SMC at both time-points of the GDS score was significantly lower (2.39 ± 2.2 before vs. 2 ± 1.8 after two years) in comparison to subjects who reported SMC at both time-points (3.62 ± 2.8 before vs. 3.62 ± 2.7 after two years) (*p* = 0.04). Moreover, among participants who did not report SMC before and reported SMC after two years, the GDS score was significantly lower (2.3 ± 3.3 before vs. 1.95 ± 2.4 after two years) in comparison to subjects who reported SMC at both time-points (3.62 ± 2.8 before vs. 3.62 ± 2.7 after two years) (*p* = 0.04) (Figure 4).

## 4. Discussion

In the above study, higher depression symptom severity at baseline assessment was associated with a higher risk of both SMC and objective decline of short-term verbal memory. This finding was consistent with a recent analysis, where depression was shown to be related to an increase in SMC [9]. In addition, in a similar study, the authors concluded that perception of decline of memory functioning rather than current memory functioning might be a significant predictor of increase in depressive symptoms in older adults [4]. In addition, previous studies revealed the same association, i.e., that SMC at baseline increases the risk of increased depressive symptom intensity [5,7].

Subjective memory complaints might occur more frequently in people experiencing negative mood [33,34]. In contrast, deficits in memory and attention are often associated with depression [35,36]. Balash et al. noted that SMC could be related to sub-syndromal depression and anxiety in cognitively intact older subjects [37]. Recent findings also indicate that depression severity might occur both before and after objective cognitive decline is noted [38].

Schmand et al. reported that SMC indicates “realistic” self-observation of cognitive decline. However, our results indicate that SMC was not significantly related to changes in short-term verbal memory test [39]. On the other hand, our results support those of a study conducted on a population from Germany, which confirmed that SMC is more strongly related to depression than objective memory decline [40].

In our study, 37 subjects (18%) reported SMC at baseline, however, were free of SMC after 2 years. Therefore, one can speculate that SMC could be transient in some older patients. In line with the above results, in a longitudinal study on 543 subjects from a rural Chinese population, 265 (49%) answered “yes” to the question “Do you have trouble with your memory?” at the baseline and 209 (39%) answered the same after 3 years [41]. It seems important to conduct further studies to explore the relationship between SMC with objective memory decline and depression severity. SMC is one of the components of a Mild Cognitive Impairment (MCI) diagnosis proposed by Petersen et al. [42]. However, some authors suggest that SMC might be more related to emotional disturbance rather than objective cognitive impairment [43], while some even strongly suggest that SMC should be excluded from the MCI criteria [44]. Therefore, further studies on mechanisms underlying SMC are needed. Moreover, understanding the underlying background of SMC might improve treatment of comorbidities of older patients with SMC.

One of the potential limitations of our study was that it used a questionnaire to capture the subjective memory complaints at only two time-points, without specifying the exact time-frame. Moreover, complaints in other cognitive domains and associated worries were not examined. One factor that could affect both the frequency and usability of an SMC is how it is measured [45]. Questions could be focused on current memory performance assessment, comparison of performance with peers, or reflect on whether the memory deteriorated over time [46]. Research examining how older adults interpret memory performance questions shows that these different types of questions do actually assess different aspects of perception of issues with memory [47,48]. Moreover, further studies should incorporate more time-points to fully reveal the relationship between the dynamics of SMC and depression intensity. In addition, further longitudinal studies on the relationship between SMC, objective memory decline and depression severity, should incorporate bigger sample sizes and examine the effects of comorbidities and treatment on this relationship. It seems that side-effects from certain groups of pharmacological agents could be associated with episodic memory decline [49]. On the other hand, deep brain stimulation in patients with Parkinson Disease presumably could lead to short-term improvement in depression [50] On the other hand, the above study is the first in Polish population to examine the relationship between depression, subjective, and objective memory decline in a longitudinal manner.

## 5. Conclusions

Our results suggest that groups of older people with SMC and objective short-term verbal memory decline do not fully overlap each other. Participants with SMC after two years were older, had a worse executive function, showed higher depression severity, a slower gait, and worse aerobic capacity.

Older people without SMC for longer than two years showed less intensity of depression symptoms in comparison to a subgroup with SMC for longer than two years. However, the occurrence of SMC in older people who were previously free of SMC, was not related to an increase in depression symptoms intensity.

## Figures and Tables

**Table 1:** Comparison of lost to follow-up vs. re-examined subjects.

Variable	Not Examined in the Second Wave (*n* = 202) Mean ± SD	Re-Examined (*n* = 205) Mean ± SD	*z* or *t*	*p*-Value
Age (years)	69.84 ± 6.3	69.66 ± 6.0	0.01	0.99
Age of memory impairment (years)	64.75 ± 7.3	65.12 ± 7.7	−0.96	0.34
MMSE (points)	27.33 ± 2.4	27.80 ± 2.1	−2.24	0.03
MoCA (points)	22.74 ± 3.7	23.63 ± 3.5	−2.46	**0.01**
MoCA Delayed Recall (words)	2.26 ± 1.7	2.25 ± 1.6	0.10	0.92
MoCA DR in overall (words)	4.62 ± 0.8	4.76 ± 0.5	−1.31	0.19
MoCA Verbal Fluency (words)	12.02 ± 4.4	12.73 ± 4.5	−1.59	0.11
TMT B (seconds)	162.74 ± 99.4	136.90 ± 88.5	3.33	**<0.01**
GDS (points)	3.37 ± 2.8	3.23 ± 2.8	0.71	0.48
Health self-assessment-currently (points)	6.73 ± 1.6	7.05 ± 1.5	−1.75	0.08
Health self-assessment-10 years ago (points)	8.09 ± 1.7	8.00 ± 2.0	0.03	0.97
Years of education (years)	13.55 ± 3.1	14.37 ± 3.4	−2.24	**0.02**
Mental and social activity lvl (points)	40.06 ± 8.5	42.72 ± 8.2	−3.05	**<0.01**
Physical activity lvl (points)	19.48 ± 7.9	20.06 ± 7.2	−0.74	0.46
Touristic activity lvl (points)	6.42 ± 3.6	6.44 ± 3.4	−0.21	0.83
Sum of rich in antioxidants food intake (points)	22.91 ± 6.2	24.11 ± 5.7	−1.23	0.22
Ethanol intake (points)	4.27 ± 3.6	4.76 ± 3.5	−1.41	0.16
Total cognitive + physical + touristic activity lvl (points)	65.95 ± 14.9	69.22 ± 14.2	−2.24	**0.02**
Financial status (points)	10.95 ± 5.3	13.12 ± 5.3	−3.96	**<0.01**

MMSE—Mini–Mental State Examination; MoCA—Montreal Cognitive Assessment; MoCA Delayed Recall—number of word correctly recalled without examiner help in the short-term verbal memory test; MoCA DR in overall—number of words correctly recalled after category and list cues in Delayed Recall subtest of MoCA, TMT B -Trail Making Test Part B, GDS—Geriatric Depression Scale; *p*-values lower than 0.05 are bolded.

**Table 2:** Comparison of SMC prevalence at the baseline and after 2 years.

SMC Baseline	SMC after 2 Years	Count (*n*)	Frequency (%)
yes	yes	125	61
no	yes	20	9.8
yes	no	37	18
no	no	23	11.2

SMC—Subjective memory complaints presence.

**Table 3:** Difference in variables measured in the baseline between subgroups with subjective memory complaint (SMC) present vs. non-present after two years.

Variable	Subjective Memory Complaint Present				*z*	*p*-Value
	Present (*n* = 162)		Not Present (*n* = 43)			
	Mean	Std. Dev.	Mean	Std. Dev.		
Age (years)	70.25	6.1	67.44	5.2	2.67	**0.01**
Years of education (years)	14.23	3.5	14.91	3.2	−1.56	0.12
MMSE (points)	27.69	2.2	28.21	1.7	−1.35	0.18
MoCA (points)	23.67	3.6	23.51	3.1	0.63	0.53
MoCA Verbal Fluency (words)	12.67	4.5	12.95	4.7	*t* = −0.36	0.72
MoCA Delayed Recall (words)	2.28	1.6	2.12	1.5	0.63	0.53
TMT B (seconds)	142.66	89.3	115.33	82.7	2.33	**0.02**
GDS (points)	3.46	2.8	2.35	2.7	2.69	**0.01**
8 ft test (seconds)	6.14	2	5.58	2	2.19	**0.03**
6MWT (meters)	496.95	97.9	535.33	105.8	−2.54	**0.01**

MMSE—Mini-Mental State Examination; MoCA—Montreal Cognitive Assessment; MoCA Delayed Recall—number of word correctly recalled without examiner help in the short-term verbal memory test; TMT B—Trail Making Test Part B; GDS—Geriatric Depression Scale, 8 ft test—8 ft up-and-go—8 ft test; and 6MWT—Six-minute walk test; *p*-values lower than 0.05 are bolded.

**Table 4:** Difference in variables measured in the baseline between subgroups with SMC present vs. non-present after two years.

Variable	Subjective and Objective Memory Impairment				*z*	*p*-Value
	Present (*n* = 60)		Not Present (*n* = 145)			
	Mean	Std. Dev.	Mean	Std. Dev.		
Age (years)	70.38	6	69.37	6	−1.20	0.23
Years of education (years)	13.55	3.3	14.71	3.4	2.54	**0.01**
MMSE (points)	27.78	2.2	27.81	2.1	−0.28	0.78
MoCA (points)	24.68	3.2	23.20	3.6	−2.74	**0.01**
MoCA Verbal Fluency (words)	12.53	4.4	12.81	4.6	*t* = 0.4	0.69
MoCA Delayed Recall (words)	3.22	1.2	1.85	1.6	−5.55	**<0.0001**
TMT B (seconds)	158.78	101.1	127.78	81.3	−2.02	**0.04**
GDS (points)	3.72	2.9	3.03	2.8	−1.71	0.09
8 ft test (seconds)	6.15	2.1	5.97	2	−0.74	0.46
6MWT (meters)	495.68	93.8	508.42	103.2	0.53	0.59

MMSE—Mini-Mental State Examination; MoCA—Montreal Cognitive Assessment; MoCA Delayed Recall—number of word correctly recalled without examiner help in the short-term verbal memory test; TMT B—Trail Making Test Part B; GDS—Geriatric Depression Scale, 8 ft test—8 ft up-and-go—8 ft test; and 6MWT—Six-minute walk test. *p*-values lower than 0.05 are bolded.

## Data Availability

Individual data are available from the corresponding author S.K. on request.
